# Role of matrix metalloproteinases in tumour invasion: immunohistochemistry of peritoneum from peritoneal carcinomatosis

**DOI:** 10.1007/s12032-018-1122-7

**Published:** 2018-04-05

**Authors:** Peter Falk, Andreas Jonsson, Torbjörn Swartling, Dan Asplund, Marie-Lois Ivarsson

**Affiliations:** 1 Fibrinolysis Laboratory/Tissue Centre, Department of Surgery, Institute of Clinical Sciences, Sahlgrenska Academy, University of Gothenburg, Sahlgrenska University Hospital/Östra, Diagnosvägen 11, 416 50 Göteborg, Sweden; 2 Varberg Hospital, Varberg, Region Halland Sweden

**Keywords:** Colorectal neoplasm, Peritoneum, Peritoneal neoplasm, Matrix metalloproteinase

## Abstract

Colorectal cancer is one of the most common forms of cancer. Spread of tumour to the peritoneal cavity may lead to seeding of cancer cells that adhere to and invade the peritoneal membrane causing peritoneal carcinomatosis. Matrix metalloproteinases (MMPs) play an essential role in cancer cell invasion and dissemination. The aim of this study was to evaluate the morphology and presence of matrix metalloproteinases in peritoneal carcinomatosis. Biopsy samples of the parietal peritoneum were taken from patients undergoing cytoreductive surgery for peritoneal carcinomatosis. The samples were fixed in formalin, dehydrated and embedded in paraffin prior to cutting into 4-µm slices. Staining with haematoxylin/eosin was used for morphology studies, and MMP-1, MMP-2 and TIMP-1 levels were evaluated using immunohistochemistry and light microscopy. The microscopically tumour-free areas of the peritoneal membrane were thin compared to the peripheral invasion zone and the areas invaded by tumour. Peritoneum invaded by tumour was richly vascularised and contained inflammatory cells. MMP-1 was expressed in tumour-free peritoneum and in the invasion zone between tumour and peritoneal tissue, but not in tumour-invaded areas. MMP-2 and TIMP-1 were mostly expressed in the proximity of blood vessels and inflammatory cells in tumour-invaded areas, but was not seen in tumour-free areas. MMPs play an important role in the process of cancer cell invasion of the peritoneum in peritoneal carcinomatosis. The peripheral zone of the tumour appears to be of importance for tumour invasion.

## Introduction

The peritoneal membrane is a pivotal organ, lining the inside of the abdominal cavity, having a surface area of approximately 2 m^2^ in an adult person [1, 2]. It consists of a monolayer of mesothelial cells lying on a basement membrane. Free colorectal cancer cells may be present in the peritoneal cavity, especially in locally advanced tumours (T4). The primary target for seeded colorectal cancer cells is the mesothelial layer lining the basal membrane of peritoneum. Thus, increasing the risk of cancer cells adhering to the peritoneal membrane and causing peritoneal carcinomatosis (PC) [3]. Earlier studies found that 5–10% of all colorectal cancer (CRC) patients develop PC in the absence of systemic disease [4, 5], while others have observed numbers close to 20% [6]. PC has traditionally been regarded as a terminal disease with a short median survival time. Since the development and application of cytoreductive surgery (CRS) and hyperthermic intraperitoneal chemotherapy (HIPEC), an aggressive and complex intervention, survival times have improved [7, 8]. However, treatment continues to be both time- and resource-consuming, has high morbidity and can only be performed at certain clinics and on selected patients.

The cancer invasion process is complex and involves the degradation of the peritoneal basal membrane. Proteolytic enzymes such as zink- and calcium-dependent matrix metalloproteinases (MMPs), produced by both tumour cells and the surrounding stroma, play an important role in this process [9]. One function is to regulate the release of other bioactive molecules such as growth factors, chemokines and adhesion molecules [10]. In a variety of cancers, activation and increased expression of MMPs generally lead to the hallmarks of tumour progression, including angiogenesis, invasion and metastasis, and high MMP level correlates to reduction in survival [11].

MMP-1 is a collagenase that degrades the extracellular matrix (ECM) of the interstitial stroma, in particular collagen Types I, II and III. In CRC, increased MMP-1 expression correlates with advanced colon cancer stage and poor prognosis [12].

MMP-2 and MMP-9 belong to the gelatinase subfamily of MMPs, and even though the main substrates for these enzymes are collagen Type IV and gelatin, they share proteolytic activity against other ECM molecules as well. Many studies on CRC have revealed a correlation between increased MMP-2 and MMP-9 expression and poor outcome. Even increased MMP-2 expression in plasma has been shown to be associated with lymph node metastasis in patients with CRC compared to those without [13].

**Fig. 1:**
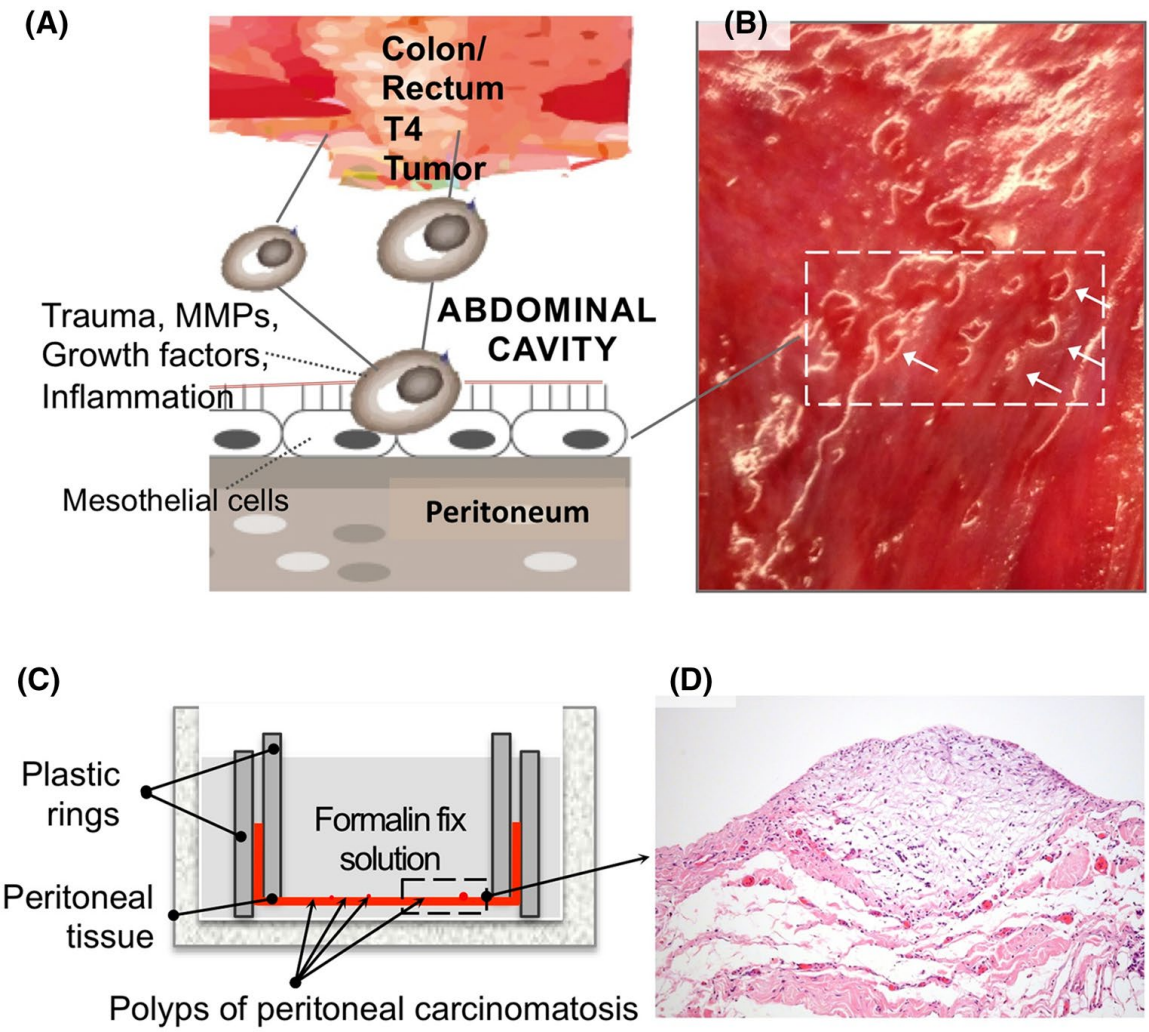
**a** A hypothetical model of the peritoneal surface being invaded by cancer cells from a colorectal tumour. Trauma, MMPs, growth factors and inflammation interact during tumour invasion. **b** Human peritoneum from a cytoreductive surgical biopsy sample showing peritoneal carcinomatosis. Accumulations of tumour cells (white arrows) have invaded the mesothelial surface on the peritoneal membrane. **c** Fixation of tumour-invaded peritoneal tissue. The peritoneal tissue was fixed overnight suspended on sterile plastic rings and submerged in 4% phosphated formalin solution. **d** Light microscopy of slices of paraffin-embedded peritoneal tissue from peritoneal carcinomatosis stained with haematoxylin/eosin. A tumour cell can be seen invading the peritoneal tissue

The invasion process is multifactorial and includes factors such as inflammation, the magnitude of surgical trauma, and the presence of ECM-degrading proteases such as matrix metalloproteinases (MMPs) and their inhibitors (TIMPs) (Fig. 1a). However, despite our knowledge of the factors involved, we know little of the biological interactions involved in terms of tissue remodelling and repair. By improving our knowledge of ECM remodelling in peritoneal tissue, and combining this with investigations on peritoneal tissue invaded by tumour cells, we aim to improve our understanding of the pathological processes involved in peritoneal carcinomatosis.

The aim of this study was to investigate matrix metalloproteinase expression in biopsy samples taken from patients with peritoneal carcinomatosis of colorectal origin.

## Materials and methods

### Patient material

During 2015, three patients undergoing total peritoneal resection by cytoreductive surgery (CRS) where included in the study after having given informed consent. The CRS procedure includes the resection of all or a major part of the parietal peritoneal membrane, with large amounts of tissue being removed. For the purposes of this study, smaller tissue samples (50 mm × 50 mm) where gently dissected away in the operation theatre. Care was taken not to damage the surface and to place the sample with the mesothelial side upwards. Biopsy samples were taken from areas with peritoneal carcinomatosis including the peripheral zone with invading cancer cells, as well as macroscopically tumour-free peritoneal tissue (Fig. 1b).

### Tissue handling and morphology

Peritoneal tissue samples were placed in a sterile bowl with the mesothelial side upwards and transported to the laboratory. Extraperitoneal fat was immediately dissected away and the resulting specimens cut into squares approximately 25 mm × 25 mm. These were then mounted between two acrylic rings facing the mesothelial side up and submerged in 4% phosphate-buffered formalin solution (Apoteket AB, Solna, Sweden) during fixation (Fig. 1c). Tissues were dehydrated in increasing concentrations of alcohol (70–99.5%) and finally made clear using xylene (Histolab, Askim, Sweden). Paraffin-embedded tissue was cut into 4-µm slices using a Mikrom HM 355 (Thermo Fisher Scientific, Waltham, MA, USA). The slices were stained for morphology studies using Mayer’s haematoxylin and eosin and examined using an Eclipse E800 (Nikon Instruments Europe, Amsterdam, Netherlands) research microscope as described previously [14] (Fig. 1d).

### Immunohistochemistry

Formalin-fixed and paraffin-embedded 4-µm tissue slices were mounted on adhesion slides (Superfrost plus, Histolab, Askim, Sweden), deparaffinised, rehydrated in xylene/alcohol gradients (99.5–70%) and rinsed twice in 5 mM Tris-buffered saline solution (TBS), pH 7.8 (Sigma-Aldrich, St Louis, MO, USA). The preparations were heated for 15 min at 95 °C in 0.01 mol/L citrate buffer, pH 6.1 as target retrieval solution (S1700; DakoCytomation, Glostrup, Denmark). Following rinsing with TBS, the preparation slides were mounted on Shandon coverplates (Thermo Fisher Scientific, USA). Non-specific protein binding was initially done using 5% fat-free dry milk in TBS, followed by endogenous peroxidase blockade using parts from the Dual Link System-HRP kit (K4065; DakoCytomation, Denmark).

Monoclonal mouse anti-human MMP-1 was diluted to 1 µg/mL (#IM35L; Calbiochem, Cambridge, MA, USA), MMP-2 diluted to 2 µg/mL (#IM51L; Calbiochem, USA) and TIMP-1 diluted to 4 µg/mL (clone 147-6D11; Calbiochem, USA) in 5% fat-free dry milk in TBS. As a negative control, monoclonal mouse antibodies of isotope IgG (X-0931, Dako, Denmark), the specificity of which is directed towards an enzyme that is neither present nor inducible in mammalian tissues was used.

Following incubation with peroxidase labelled polymer conjugated to goat anti-mouse immunoglobulin, the sections were incubated using 3,3′-diaminobenzidine (DAB+) as a chromogenic substrate generating a brownish colour indicating a positive signal, all according to the manufacturer’s instructions (Dual Link System-HRP, Dako, CA, USA).

After rinsing with TBS, counterstaining was performed using Harris HTX and mounting achieved with Pertex (Histolab, Sweden) following dehydration using alcohol (70–99.5%) and xylene as previously described. Similar techniques have been described previously [14, 15].

Immunohistochemical evaluation was performed by photographic documentation using the Eclipse E800 microscope connected to CoolPix 995 photographic equipment (Nikon, Netherlands). Immunohistochemical staining was performed in several serial dilutions using duplicates for each dilution.

## Results

### Morphology and histological evaluation

**Fig. 2:**
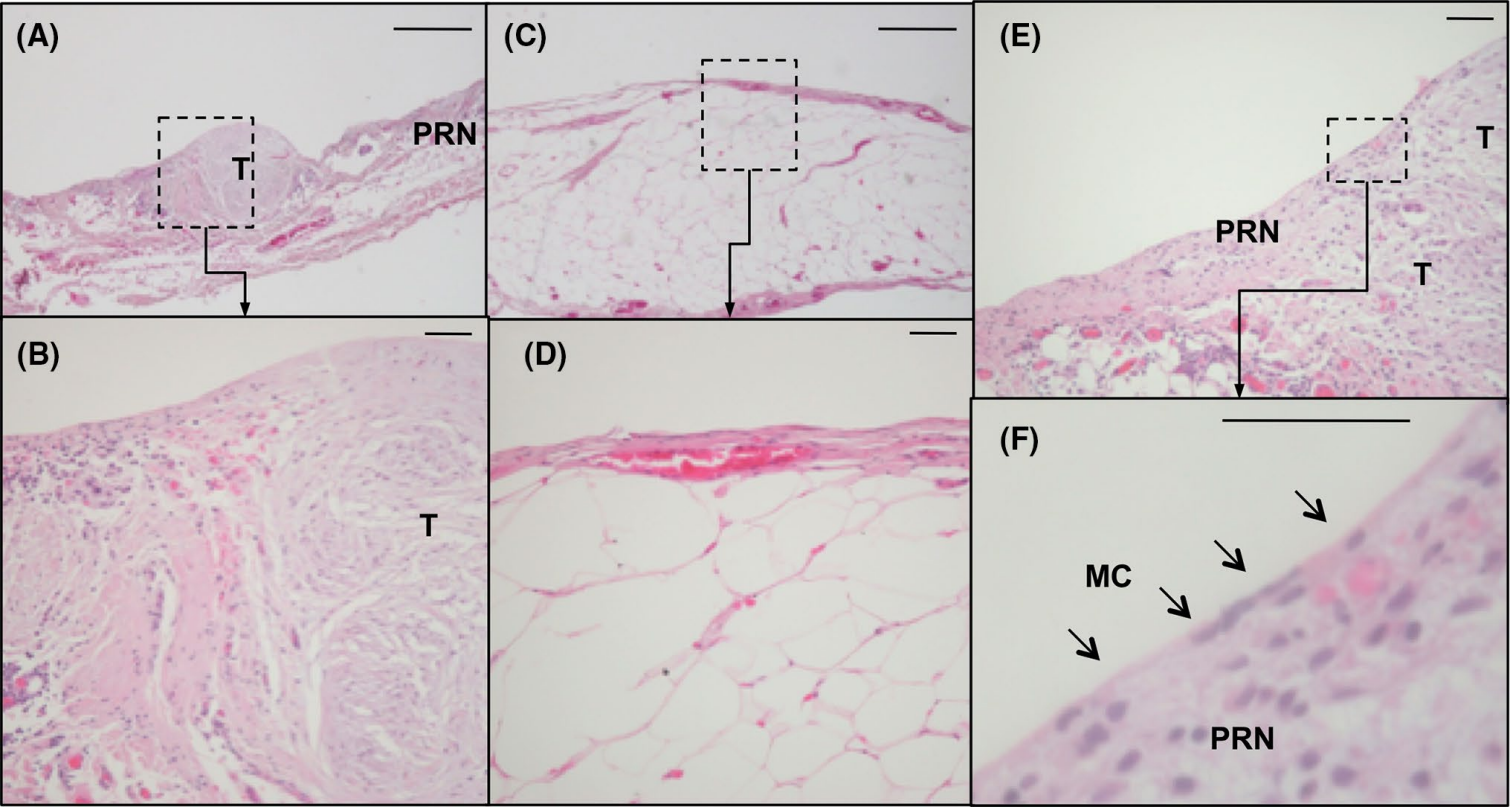
Micrographs of human peritoneum invaded by tumour compared to tumour-free peritoneum from the same patient. The structure and thickness of the peritoneal membrane differs between the tumour-invaded areas (**a**, **b**) and the tumour-free areas of the peritoneal membrane (**c**, **d**). Single mesothelial cells are covering not only the tumour-free peritoneum, but also the tumour tissue invading the peritoneal surface (**e**, **f**). Haematoxylin/eosin stained paraffin-embedded peritoneum taken during cytoreductive surgery for peritoneal carcinomatosis. Size marker 500 µm (**a** + **c**) and 50 µm (**b** + **d** − **f**), T = tumour, PRN = peritoneum, MC = mesothelial cells

Histologically, the macroscopically tumour-free peritoneal tissue differed from tissue invaded by cancer cells in several respects. The peritoneum in close proximity to the tumour was thicker and had more vessels and inflammatory cells than the microscopically tumour-free areas of the peritoneal membrane. Peritoneal tissue invaded by cancer had a more rigid and fibrotic appearance in the mesothelial and submesothelial surface, compared to tumour-free peritoneum (Fig. 2).

A thin layer of mesothelial cells was seen covering not only the tumour-free peritoneum, but also areas of peritoneum invaded by tumour (Fig. 2e–f).

### Inflammation and vascularisation

**Fig. 3:**
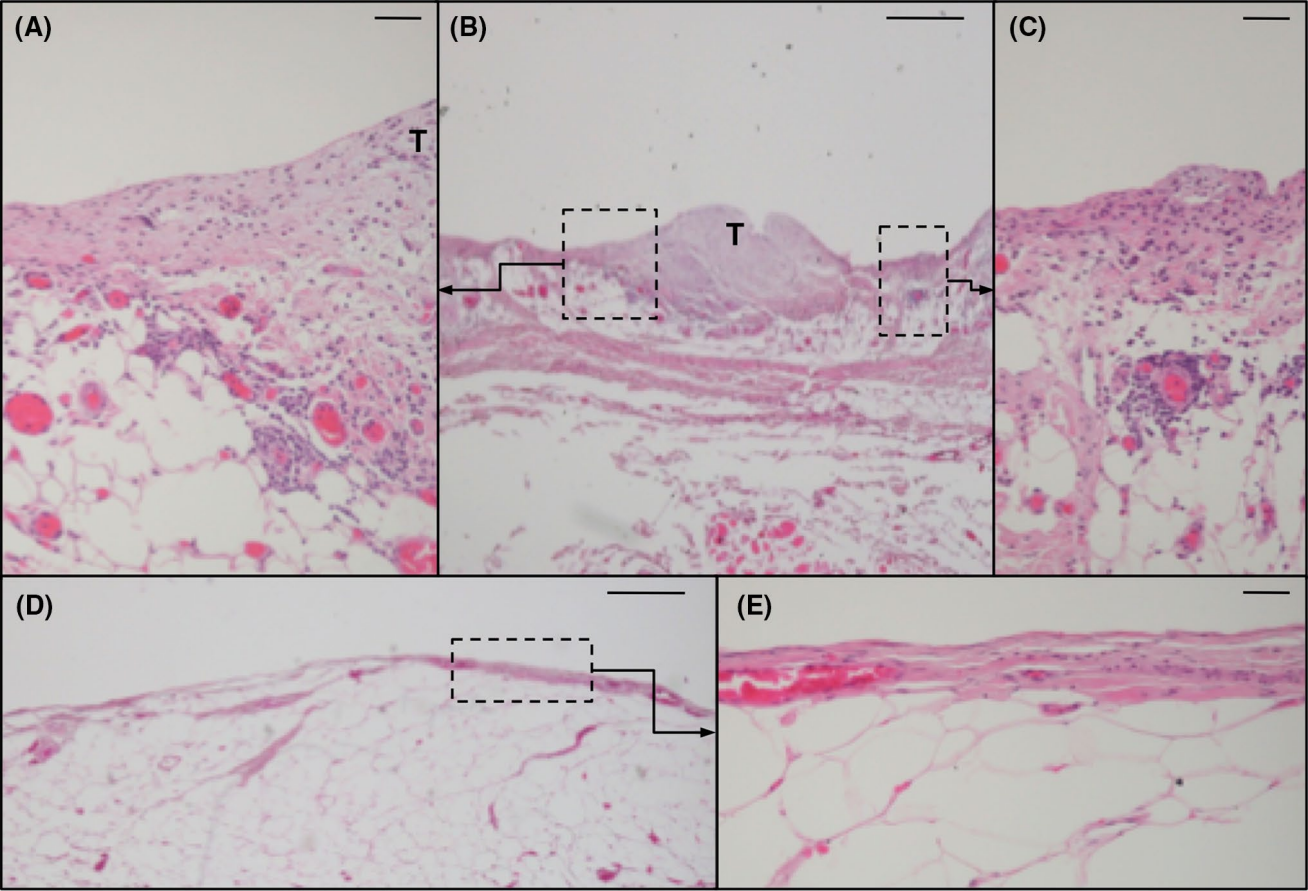
Micrographs of human peritoneum invaded by tumour compared to tumour-free peritoneum. The presence of inflammatory cells and vascularisation is much greater in the tumour-invaded peritoneum (**a**–**c**) compared to tumour-free areas (**d**, **e**). Haematoxylin/eosin stained paraffin-embedded peritoneum taken during cytoreductive surgery for peritoneal carcinomatosis. Size marker 500 µm (**b** + **d**) and 50 µm (**a**, **c**, **d**), T = tumour, PRN = peritoneum

Morphological evaluation of micrographs of the Htx/Eos stained slices showed inflammatory cells and rich vascularisation in the peritoneal areas invaded by cancer cells and in the immediate adjacent area. This is in contrast to the tumour-free peritoneal membrane where only limited signs of inflammation were seen (Fig. 3).

### Presence of matrix metalloproteinases

#### MMP-1

**Fig. 4:**
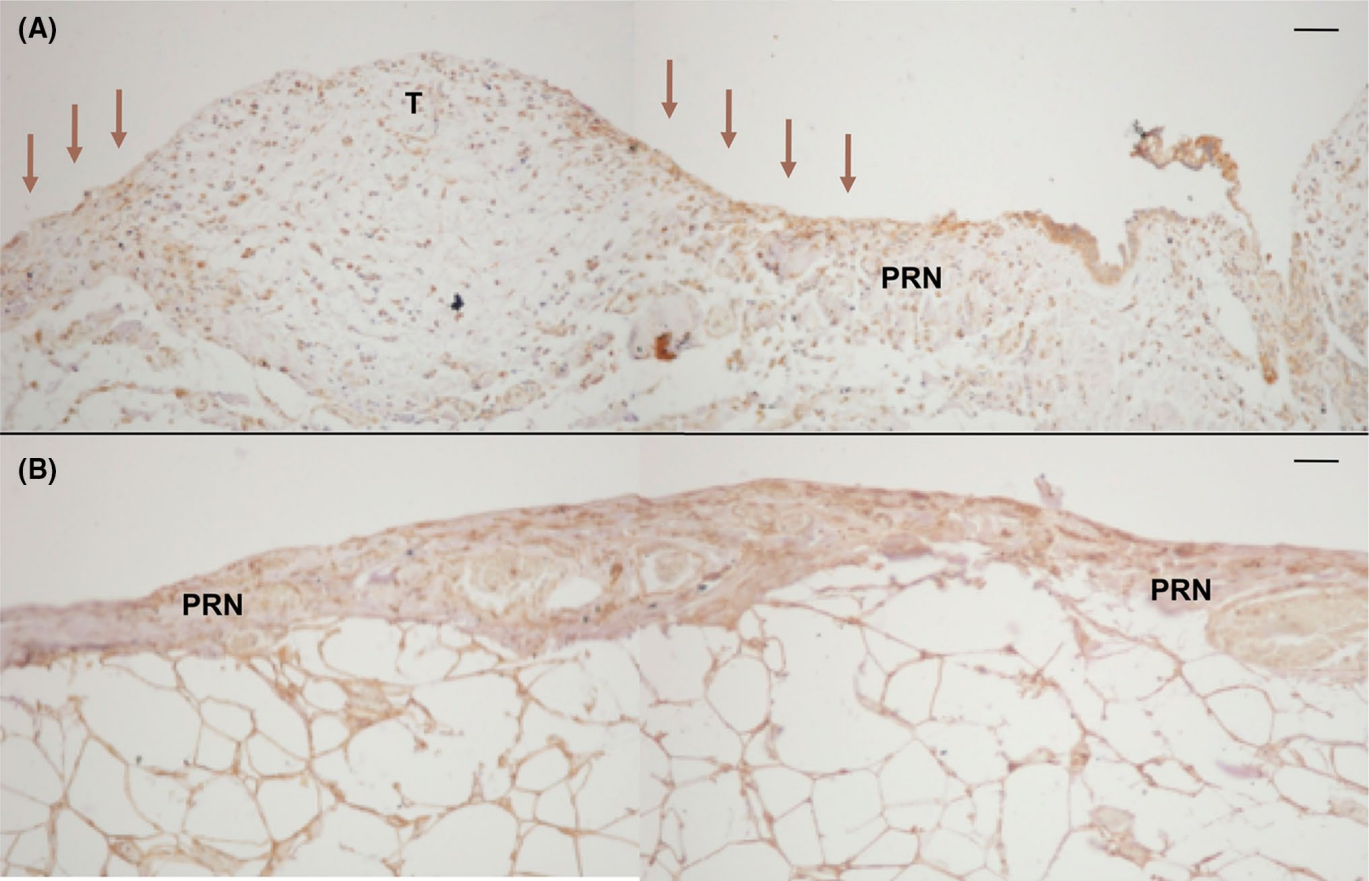
Matrix metalloproteinase 1 (MMP-1) expression in human tumour-invaded peritoneum compared to tumour-free peritoneum. Expression of MMP-1 in the tumour-invaded peritoneal tissue was generally seen in the invasion zone between the tumour and normal peritoneal tissue (**a**, arrows). In the microscopically tumour-free areas, a more general expression of MMP-1 staining was seen (**b**). Dako Enviosion with brownish diaminobenzidine (DAB) counterstained with haematoxylin on paraffin-embedded peritoneum taken during cytoreductive surgery for peritoneal carcinomatosis. Size marker 50 µm (**a**, **b**), T = tumour, PRN = peritoneum

Matrix metalloproteinase 1 (MMP-1) expression in tumour-invaded peritoneum was more intense in the peripheral invasion zone between the tumour and normal peritoneal tissue, indicating an active role of MMP-1. In the microscopically tumour-free areas, a more general expression of MMP-1 was seen (Fig. 4).

#### MMP-2

**Fig. 5:**
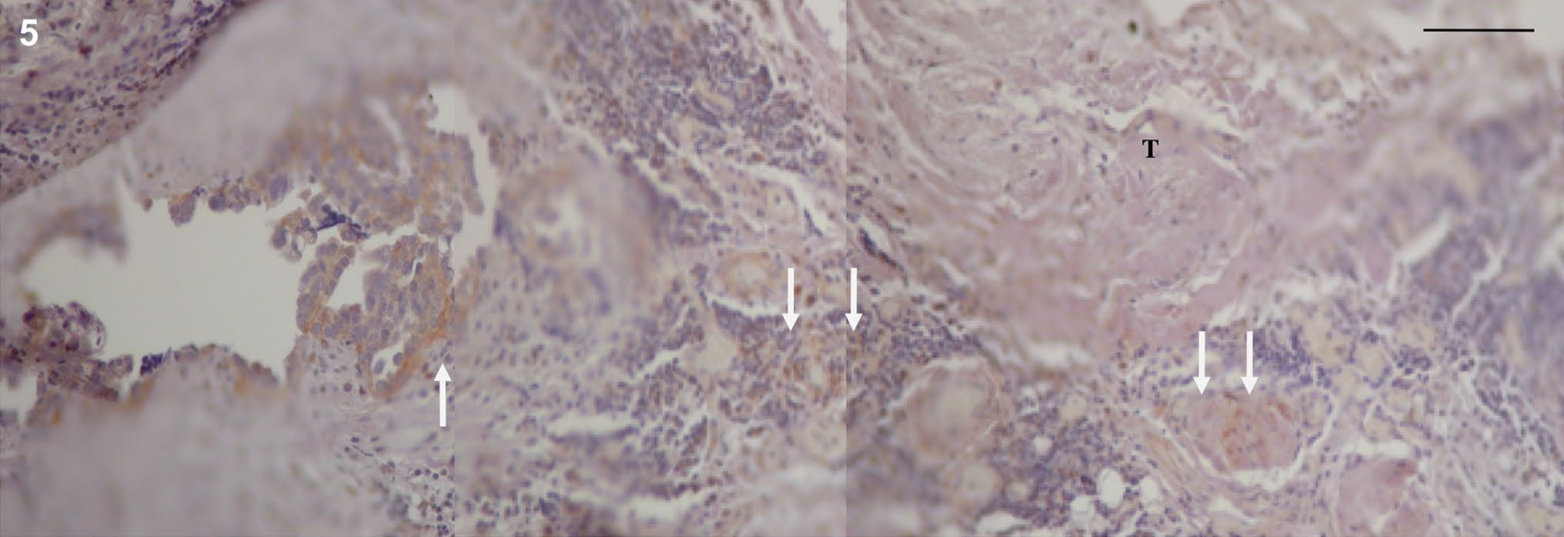
Matrix metalloproteinase-2 (MMP-2) expression in human tumour-invaded peritoneum compared to tumour-free peritoneum. In the tumour-invaded peritoneal tissue, expression of MMP-2 was only seen in the proximity of inflammatory cells (arrows) and vascular structures (arrows). No expression was seen in the invasion zone of the tumour or microscopically tumour-free areas of the peritoneal tissue (data not shown). Dako Enviosion with brownish diaminobenzidine (DAB) counterstained with haematoxylin on paraffin-embedded peritoneum taken during cytoreductive surgery for peritoneal carcinomatosis. Size marker 50 µm, T = tumour

Matrix metalloproteinase-2 (MMP-2) expression in the tumour-invaded areas was only seen in the proximity of inflammatory cells and blood vessels (Fig. 5). No expression was seen in the peripheral zone or in the microscopically tumour-free areas of the peritoneal tissue (data not shown).

#### TIMP-1

**Fig. 6:**
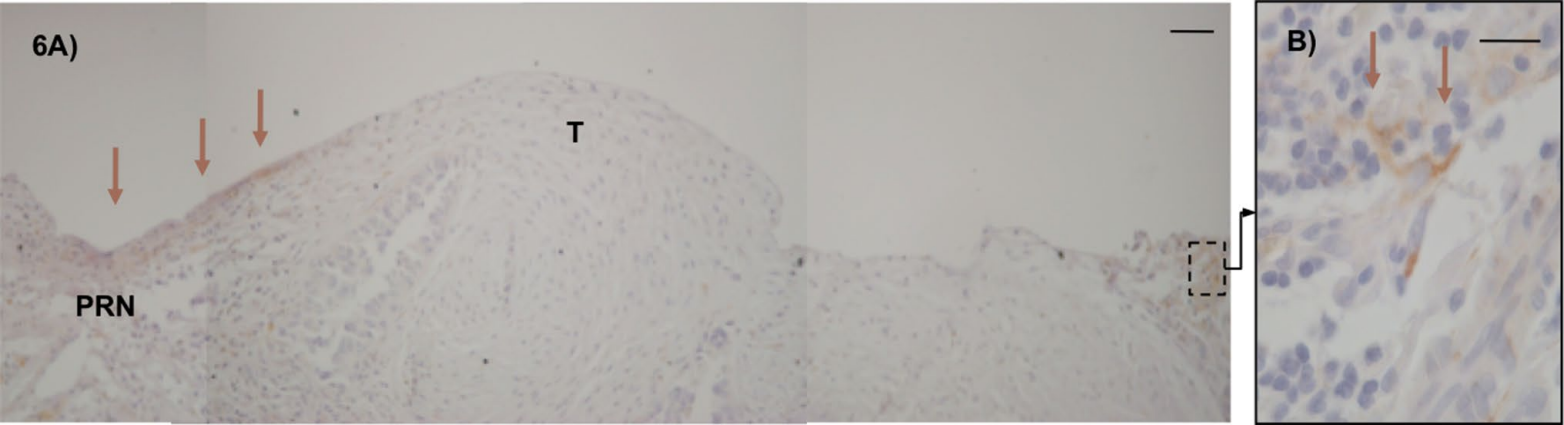
Tissue inhibitor of metalloproteinase-1 (TIMP-1) expression in human tumour-invaded peritoneum compared to tumour-free peritoneum. In the tumour-invaded peritoneum, TIMP-1 expression was only sparsely seen in the invading zone between the tumour and peritoneal tissue (**a**, arrows) and in the proximity of inflammatory cells (**b**, arrow). No expression was seen in the microscopically tumour-free areas (data not shown). Dako Enviosion with diaminobenzidine (DAB) counterstained with haematoxylin on paraffin-embedded peritoneum taken during cytoreductive surgery for peritoneal carcinomatosis. Size marker 50 µm (**a**) and 20 µm (**b**), T = tumour, PRN = peritoneum

Tissue inhibitor of metalloproteinase-1 (TIMP-1) expression in peritoneum invaded by tumour was only sparsely seen in the invasion zone between the tumour and normal peritoneal tissue and close to the inflammatory cells present (Fig. 6). No expression was seen in the microscopically tumour-free areas of the peritoneal tissue (data not shown).

## Discussion

Peritoneum covers the inside of the abdominal wall as well as the internal organs. A monolayer of mesothelial cells lining the basal membrane of the peritoneum [1, 2]. When colorectal cancer, primarily T4 tumours, spreads to the abdominal cavity, the mesothelial cell layer is a natural target for seeding cancer cells. These spread to the peritoneal surface and become attached by anchorage and adhesion to the mesothelial surface, leading to peritoneal carcinomatosis. A combination of CRS and HIPEC has been used to treat PC [7, 8], but unfortunately this treatment is associated with high morbidity and can only be used in selected patients.

The present study investigated the morphology as well as MMP and TIMP-1 levels in normal peritoneal tissue and peritoneum invaded by cancer cells, in samples taken during CRS for peritoneal carcinomatosis.

We demonstrated that in peritoneal carcinomatosis, microscopically tumour-free peritoneal tissue differs from peritoneal tissue invaded by tumour cells. The peritoneum of tumour-free areas was thinner than the peripheral zone of the tumour-invaded tissue and the areas invaded by tumour. The peritoneal membrane is a highly dynamic organ and is able to adapt in both structure and function [16]. It has a key role in regulating inflammatory processes and the exchange of peritoneal fluid, thereby preventing fibrosis in the abdominal cavity. A regulatory imbalance affects the environment in the abdominal cavity with ascites, fibrotic adhesions and inflammation, all of which promote the development of PC [16].

We were also able to demonstrate the presence of inflammatory cells in the region where tumour had invaded the peritoneal membrane. Such inflammatory cells were not present to the same extent in the tumour-free areas (Fig. 4). The establishment of PC involves a complex sequence of stages. In 2003, Jayne described how injured peritoneal membranes following abdominal surgery are rich in cytokines and growth factors that facilitate tumour proliferation and invasion [17]. Adhesion of cancer cells to the peritoneum depends on the presence of adhesion molecules and integrins expressed by inflammatory cells via an established microvascular mesh. The process of angiogenesis thus seems to be of importance, even if its role in the peritoneal metastatic cascade is not understood. Jayne et al. also described how tumour invasion of the mesothelial layer somehow leads to mesothelial apoptosis. Whether or not this is associated with the mesenchymal transition to fibrocytes described previously [18], remains to be seen.

We observed the presence of a thin layer of mesothelial cells lining not only microscopically healthy peritoneum but also peritoneum invaded by cancer. The function of a mesothelial layer lining tumour tissue is not understood. Signal transmission pathways between the peritoneal mesothelial cells and the invading tumour are complex and the mesothelial layer could have several roles.

One of these roles is that the mesothelial cells of the peritoneal membrane are unique in their ability to repair damage following trauma or surgery, as demonstrated by Mutsaers and coworkers [19]. The peritoneum maintains a fine balance between fibrotic and fibrinolytic processes. The occurrence of adhesions after abdominal surgery has been shown to be caused by a defect in fibrinolysis, demonstrating the importance of this balance [20]. The processes involved in invasion of the peritoneal surface by tumour cells are only partly understood, but these may well be similar to those following trauma or surgical damage. The fibrotic appearance could be acting as a scaffold for invading tumour cells. Whether or not the mesothelial layer covering the tumour cells in the peritoneal membrane has a role to play in this respect remains to be seen.

During normal and pathological conditions, mesothelial cells have the capacity to promote the formation and deposition of fibrin as part of the peritoneal repair process. Not only do mesothelial cells build extracellular matrix, but they also have the capacity to promote its degradation. By producing plasminogen activators and their inhibitors, mesothelial cells are important in the conversion of plasminogen to plasmin causing local fibrin degradation and a general regeneration in tissue repair [21]. Matrix metalloproteinases (MMPs) are produced by the peritoneal membrane and have the capacity to degrade the extracellular matrix of the surrounding stroma [22]. Together with their inhibitors (TIMPs), these enzymes are crucial in peritoneal repair resulting in complete healing within a week after trauma, regardless of the extent of damage to the peritoneum [23, 24]. Injury in any form can disrupt the balance between MMPs and TIMPs, resulting in permanent fibrin adhesions or, in the case of PC, a microenvironment favourable for tumour invasion.

The roles played by MMPs and TIMPs in PC are not fully understood. Desmeules et al. [25] investigated MMP-2, MMP-9 and TIMP-2 expression in immunohistochemical studies on peritoneal samples from 100 women undergoing cytoreductive surgery for peritoneal carcinomatosis from ovarian cancer. The authors aimed to see whether TIMP-2, MMP-2 and MMP-9 expression could predict progression and survival. The presence of increase levels of MMP-9 was associated with poorer survival [25], but no association was seen between MMP-2 or TIMP-2 expression and prognosis. Only loss of collagen structure has been associated with high levels of both MMP-2 and MMP-9. In 1999, Zeng and coworkers demonstrated that a loss of Type IV collagenase in the extracellular matrix is associated with local increase in the levels of MMP-2 and MMP-9 [9].

In the present study, the presence of MMP-2 in tumour-invaded peritoneum was only seen in the proximity of inflammatory cells and vascular structures. No expression of MMP-2 was seen close to the tumour-invaded area or in tumour-free parts of the peritoneal tissue. The presence of TIMP-1 was only seen in the zone between tumour and peritoneal tissue and in the proximity of inflammatory cells. No expression could be seen in microscopically tumour-free peritoneal tissue samples.

The role of MMP-1 has previously been discussed in relation to splenic angiosarcoma, a cancer that can metastasise to the membranes of both the peritoneum and pleura, much like mesothelioma. Using immunohistochemical staining, the authors observed that the tumour expressed MMP-1, an enzyme known to increase the invasiveness of mesothelioma cells. The authors suggested that MMP-1 generates a fibrous stroma that aids invasion of the tumour [26]. In the present study, the expression of MMP-1 was generally seen in tumour-free peritoneum and not peritoneum invaded by tumour. Similar observations have been made previously [26]. The reason for this is not known, but it may be that peritoneal tissue undergoes a process involving MMP-1 that prepares tissues for tumour invasion. The lack of MMP-1 expression in peritoneum already invaded by tumour could possibly be explained by mechanisms that keep tissues in a remodelling state in the invasion zone between normal peritoneum and tumour. This is supported by the local presence of MMP-1 in the peripheral zone of tumour-invaded areas.

The process of tumour invasion in PC is complex and involves the degradation of the peritoneal basement membrane. Proteolytic enzymes produced by stromal, mesothelial and tumour cells play an important role in this inflammatory process, which depends, among other things, on the magnitude of surgical trauma and the presence of extracellular matrix degrading proteases such as MMPs and their inhibitors (TIMPs), although our knowledge about these biological interactions is limited.

The present work adds to our knowledge regarding the presence of increased levels of MMP-1 in PC. Peritoneal carcinomatosis involves a complicated sequence of interdependent stages, from tumour cells released from a primary tumour in the abdominal cavity that might be affected by intraoperative trauma during surgery, via ongoing inflammation and other factors, to the unique capacity of the tumour cells to invade the peritoneum. Proteases seem to play an important role in the invasion process.

## Conclusion

The study provides increased knowledge of the role of proteases in peritoneal carcinomatosis where MMP-1 appears to be of importance for cells invasion of the peritoneum. Further research investigating the components of the peritoneal metastatic cascade is needed to develop more successful therapeutic strategies for this mortal condition.
